# Assessment of a health facility based active case finding system for Ebola virus disease in Mbandaka, Democratic Republic of the Congo, June–July 2018

**DOI:** 10.1186/s12879-019-4600-4

**Published:** 2019-11-21

**Authors:** Amber Kunkel, Mory Keita, Boubacar Diallo, Olivier le Polain de Waroux, Lorenzo Subissi, Bocar Wague, Roger Molala, Pierre Lonfandjo, Sébastien Bokoo Bokete, William Perea, Mamoudou Harouna Djingarey

**Affiliations:** 1 Emerging Diseases Epidemiology Unit, Institut Pasteur, Paris, France; 2 Global Outbreak Alert and Response Network (GOARN), Geneva, Switzerland; 3 WHO Regional Office for Africa, Brazzaville, Republic of the Congo; 4 Public Health England, London, UK; 5 UK-Public Health Rapid Support Team, London, UK; 6 Department of Infectious Disease Epidemiology, London School of Hygiene and Tropical Medicine, London, UK; 7 Sciensano, Brussels, Belgium; 8 Ministry of Health of Mauritania, Nouakchott, Mauritania; 9 Provincial Health Division of the Équateur Region, Mbandaka, Democratic Republic of the Congo; 10 WHO Headquarters, Geneva, Switzerland

**Keywords:** Active case finding, Ebola, Ebola virus disease, Health facility, Surveillance, Democratic Republic of the Congo

## Abstract

**Background:**

The ninth outbreak of Ebola Virus Disease (EVD) in the Democratic Republic of the Congo occurred in Équateur Province from 8 May-24 July 2018. A system of health facility (HF)-based active case finding (ACF) was implemented in Mbandaka, a regional capital with four confirmed EVD cases, following completion of contact tracing. The goal of this HF-based ACF system was to look for undetected EVD cases among patients that visited HFs beginning one week prior to the system’s implementation.

**Methods:**

From 23 June – 24 July 2018, ACF teams visited HFs in Mbandaka and reviewed all medical records as far back as 17 June for any consultations meeting the suspected EVD case definition. The teams then assessed whether to validate these as suspected EVD cases based on factors such as recovery, epidemiological links, and their clinical judgement. ACF teams also assessed HFs’ awareness of EVD symptoms and the process for alerting suspected cases. We calculated descriptive statistics regarding the characteristics of reviewed consultations, alert cases, and visited HFs. We also used univariate and multivariate random effects logistic regression models to evaluate the impact of repeated ACF visits to the same HF on the staff’s awareness of EVD.

**Results:**

ACF teams reviewed 37,746 consultations, of which 690 met the definition of a suspected case of EVD. Two were validated as suspected EVD cases and transferred to the Ebola Treatment Unit for testing; both tested negative. Repeated ACF visits to the same HF were significantly associated with improved EVD awareness (*p* < 0.001) in univariate and multivariate analyses.

**Conclusion:**

HF-based ACF during EVD outbreaks may improve EVD awareness and reveal many individuals meeting the suspected case definition. However, many who meet this definition may not have EVD, depending on the population size covered by ACF and amount of ongoing EVD transmission. Given the burdensome procedure of testing suspected EVD cases, future HF-based ACF systems would benefit from improved clarity on which patients require further testing.

## Background

Ebola Virus Disease (EVD) is notorious for its high mortality rate and propagation in healthcare facilities (HFs). Periodic outbreaks of EVD have been recognized since 1976, most notably the 2013–2016 West African epidemic, which resulted in over 28,000 cases and 11,000 deaths [1, 2]. Multiple EVD outbreaks have occurred in the Democratic Republic of the Congo (DRC). This paper focuses on its ninth epidemic, which occurred in Équateur Province in 2018; its tenth and largest yet, centered in North Kivu and Ituri Provinces, has caused over 2500 confirmed and probable cases as of 14 July 2019, and on 17 July 2019 was declared a Public Health Emergency of International Concern [3, 4].

**Fig. 1:**
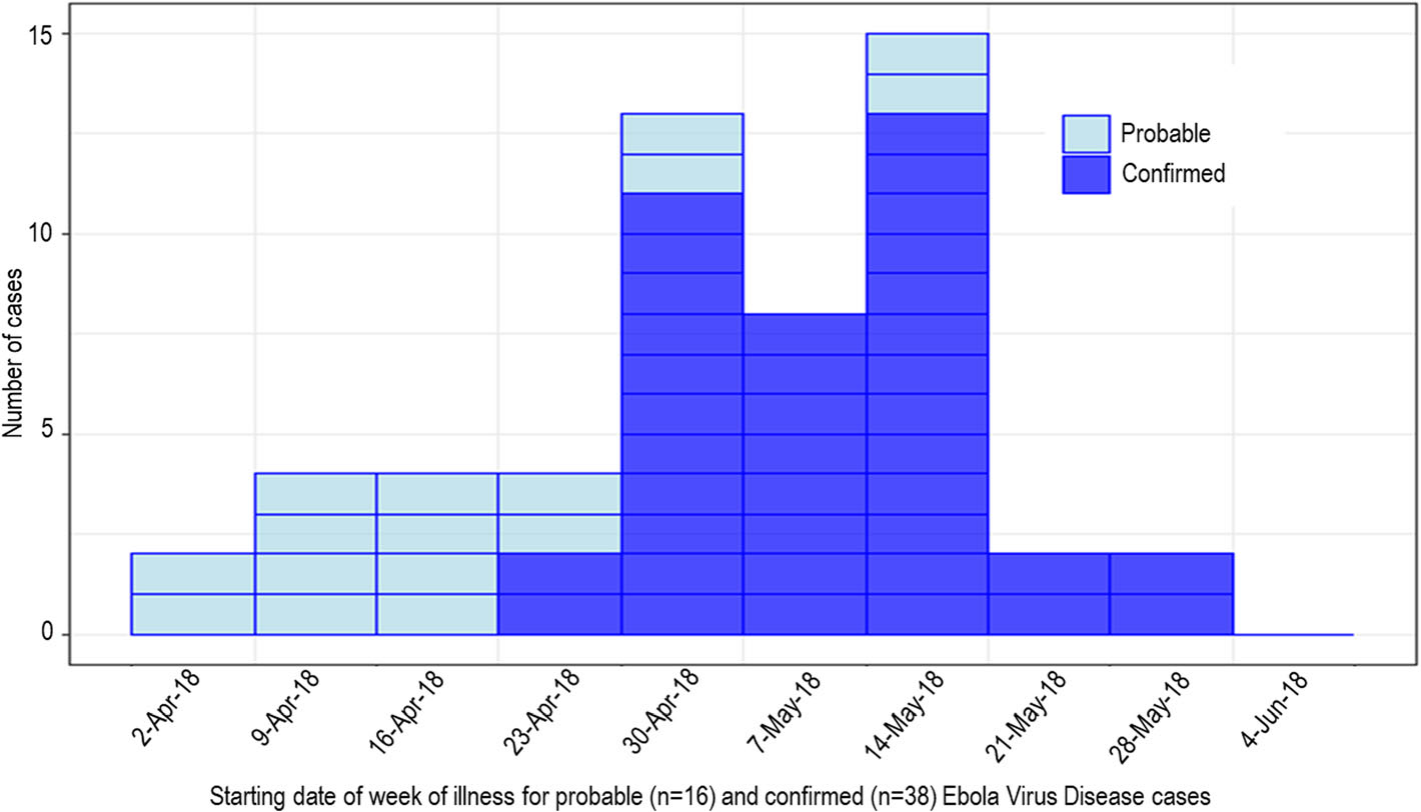
Epidemiological curve by week of illness onset, Équateur Province, Democratic Republic of the Congo, 2018 [5]

The ninth outbreak of EVD in DRC was declared on 8 May 2018 in Équateur Province. Following the outbreak declaration, the Ministry of Health (MoH) and partners quickly mounted an epidemic response. The second negative test of the final detected EVD patient occurred on 12 June, and the official end of the epidemic was declared 42 days (i.e. two incubation periods) later, on 24 July 2018. The epidemic had a total of 54 confirmed and probable EVD cases (Fig. 1), of which 33 died (overall Case-Fatality Rate: 61%) [5]. Of major concern was the occurrence of four confirmed cases in Wangata Health Zone in the city of Mbandaka, a regional transportation hub bordering the Republic of the Congo with a population of around one million people [5, 6].

EVD surveillance in the early phase of this outbreak, as in previous EVD epidemics, relied primarily on detection and follow-up of all contacts of known EVD cases. Although contact tracing can be very effective, it is important that all contacts be successfully identified and followed up [7]. As in previous outbreaks, an early warning ‘alert’ system, with alerts reported from both formal healthcare structures as well as the community, was also implemented [8]. Active case finding (ACF) is another key surveillance activity for control of EVD transmission, which relies on active early identification of suspect cases who may not otherwise be reported [9]. For example, ACF teams may visit households or HFs to look for possible EVD cases [10, 11]. Although ACF has been used in almost all previous EVD outbreaks either at the beginning, throughout, or at the tail end, published data on its impact are limited.

HFs provide an appealing location for ACF based on symptom screening. Due to the severity of symptoms, individuals with EVD are likely to come in contact with the healthcare system during the course of their illness. However, these cases may not be detected if health care workers (HCWs) lack awareness of EVD symptoms and the procedure for reporting suspected cases [12]. In previous epidemics, the occurrence of a nosocomial outbreak involving HCWs has sometimes been the event that triggered EVD detection [13, 14]. In the 2013–2016 West African EVD epidemic, nosocomial transmission to HCWs outside of Ebola treatment units (ETUs) by patients with undetected EVD continued after the establishment of these units [12, 15]. Infection in HFs has also contributed significantly to transmission in the ongoing DRC epidemic [16].

However, symptom-based surveillance for EVD is complicated by the low specificity of EVD symptoms. Studies comparing symptoms among people diagnosed with EVD and people tested negative for EVD have revealed this challenge [17, 18]. Other diseases such as malaria may be mistaken for EVD, and vice-versa [19, 20]. Furthermore, the positive predictive value of the case definition in the absence of an epidemiological link will vary based on the prevalence of EVD, which itself depends on the status of the epidemic and the size of the population being surveilled; in Mbandaka, which had very few confirmed cases and a large population size, we believed this value to be extremely low. Evidence is needed to show how well HF-based ACF for EVD works given these diagnostic challenges.

In this paper, we outline the implementation of an HF-based ACF system in Mbandaka during the 2018 Équateur EVD outbreak and discusses the strengths and weaknesses of the system put in place.

## Methods

### ACF system

The HF-based ACF system in Mbandaka took place from 23 June – 24 July 2018, following the second negative test of the final EVD patient and during the period of enhanced surveillance between the end of contact tracing and the official end of the epidemic. The objectives were 1) early detection of all potential cases of EVD in HFs, and 2) reinforcement of the previously-established system of alerts, consisting of a phone hotline that could be called by HFs, points of entry, and other key sites upon suspicion of an EVD case.

The HF-based ACF visits were conducted by MoH teams of doctors and nurses. To guide their visits, lists were created in advance of all known HFs in each of the three health zones of the city of Mbandaka. Each HF was assigned a priority based on the typical number of consultations per week and the types of medical services offered. The targeted frequency of visits was twice a week for high-priority HFs, once a week for medium-priority, and once every two weeks for low-priority, aiming to maximize the utility of a limited number of ACF teams. Thus, during the four-week period of enhanced surveillance, each high-priority HF should have been visited at least eight times, each medium-priority HF at least four times, and each low-priority HF at least twice. ACF teams could also visit any HFs that were not on this initial list that were recognized based on their knowledge of the area or discussions with others.

Upon the first ACF visit to an HF, the teams presented themselves to the HF’s head to discuss their visit and to request a designated focal point. At the start of this and all subsequent visits to the HF, the ACF team began by asking about four EVD awareness indicators: 1) knowledge of the definition of a suspected case of EVD (Table 1), 2) knowledge of the system of alerts, 3) knowledge of the phone number to call if a patient met the suspected case definition, and 4) having already posted a flyer including the phone number and definition of a suspected case in a visible area. The ACF team would then supply the HF focal point with any information missing from their responses. For the analyses presented here, HFs that responded yes to all four indicators were considered to have good EVD awareness.

**Table 1:** Definition of a suspected case of EVD used for HF-based ACF during the 2018 Equateur EVD outbreak

Definition of a suspected case of EVD	
1. Any unexplained death	
2. Any unexplained bleeding	
3. Any spontaneous abortion	
4. Fever > 38 °C and contact with a probable or confirmed case of EVD	
5. Fever > 38 °C and contact with a sick or dead animal	
6. Fever > 38 °C and ≥ 3 of the following symptoms:	Vomiting
	Diarrhea
	Intense fatigue
	Anorexia/lack of appetite
	Abdominal pain
	Muscle or joint pain
	Headache
	Difficulty in swallowing
	Difficulty in breathing
	Skin rash
	Hiccups

A person meeting any of the numbers 1–6 would meet the suspected case definition

The ACF team next examined the HF registry or consultation sheets with the HF focal point to ensure they had sufficient information to apply the definition of a suspected case of EVD and identify the patients if necessary, i.e. name, address, telephone number, symptoms, etc. The ACF team then reviewed all recorded consultations starting with the day of the current visit and moving backwards until either 17 June (one week before HF-based ACF implementation, for the first visit to an HF) or the day of the previous visit (for repeated HF-based ACF visits to the same HF). Any consultation meeting the definition of a suspected case of EVD (Table 1) was to be considered an alert case. The ACF teams discussed these cases with the HF staff to ask whether they had already been alerted; if not, they were treated as new alert cases. The ACF teams evaluated the new alert cases based on the information available at the HF, supplemented by phone interviews with the patients or household visits allowing for clinical observation by the ACF investigators. ACF investigators assessed the patients’ clinical signs and symptoms, the course of their illness, and the presence of EVD risk factors to determine whether they should be validated as EVD suspected cases and transferred to the ETU for testing and treatment. Suspected cases were reported to the alert center by telephone via EWARS (Early Warning Alert and Response System). Those not retained as suspected cases were designated as “invalidated” alert cases. There were no specific guidelines on which cases should be validated or invalidated. Rather, these assignments were subjectively made on an ad-hoc basis, with only those that the ACF investigators judged highest risk being validated. Clinical improvement was used as a reason not to validate a case; however, the diagnoses assigned by the HFs were not assumed to rule out the possibility of EVD. Information including age, sex, symptoms, and diagnosis determined by the HF was recorded for all new alert cases, both validated and invalidated. The specific reason a case was validated or invalidated was typically not recorded. Finally, the ACF team provided feedback to the HF focal point regarding the registry (such as whether it was up to date and included sufficient details, and how many cases met the suspected case definition).

#### Data management and analysis

Summaries of each HF-based ACF visit, such as the number of reviewed consultations, the number of alert cases, and responses to the EVD knowledge indicators, were recorded on paper forms that were then entered into an ACF database. Separately, details of new alert cases such as age, sex, and symptoms were recorded on paper and then entered into an alert database. The alert database also included data from new alert cases arising outside of the HF-based ACF system, and was updated daily based on the received alerts. All data were collected for surveillance and public health rather than research purposes. Prior to the analyses in this paper, the alerts and ACF data sets were evaluated for discrepancies and corrected using the data from the paper forms when possible. The master list of HFs was also improved by adding all HFs visited by ACF teams that were not already listed; these HFs were assumed to have low priority.

The ACF data set was used for all analyses related to HFs visited and consultations reviewed, whereas the alerts data set was used for all analyses describing the characteristics of alert cases. Analyses consisted primarily of descriptive statistics regarding the characteristics of reviewed consultations, alert cases, and visited HFs. Logistic regressions with a random effect for HF to account for repeated visits were run to evaluate the effect of various HF and visit characteristics on EVD awareness, with the main variable of interest being the number of previous ACF visits to the same HF. All statistical analyses were performed using R, with regression models performed using function glmer from package lme4 [21]. Details of the models used are provided in Additional file 1.

## Results

### Description of consultations reviewed

**Fig. 2:**
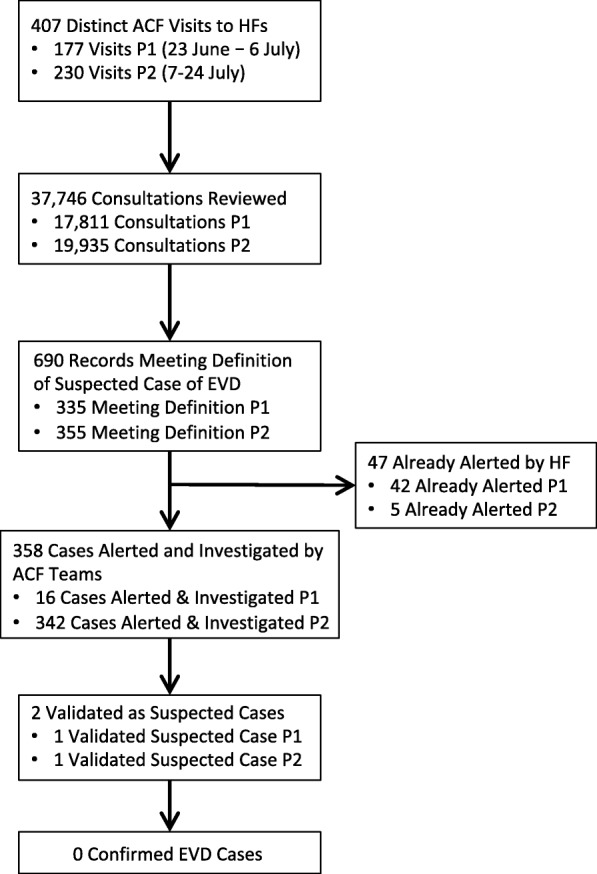
Flowchart of HF-based ACF activities. P1: Period 1, 23 June - 6 July; P2: Period 2, 7–24 July

From 23 June – 24 July 2018, HF-based ACF teams made 407 visits to 113 HFs in Mbandaka. The teams reviewed the records for 37,746 consultations occurring from 17 June – 24 July 2018, of which 690 met the definition of a suspected case of EVD, 358 were alert cases, and 2 were validated as suspected EVD cases and transferred to the ETU for testing (Fig. 2); both tested negative. One consultation meeting the definition of a suspected case of EVD was found for every 55 consultations reviewed. However, only one per 345 consultations meeting this definition was validated by investigators as a new suspected case.

For comparison, two of the 89 alert cases that were alive at the time of the alert and arose from the alert system excluding HF-based ACF from 23 June – 24 July 2018 were validated as suspected cases. Eighteen of these 89 alerts arose from points of entry (zero validated), one from the community (zero validated), two from HFs outside of the HF-based ACF zones (one validated), and 68 from HFs within the HF-based ACF zones (one validated). ACF investigators classified 47 of the alert cases that they detected as “already alerted”, suggesting they did not record all such cases. As the ACF investigators did not collect further information on these cases, we did not attempt further to link these data sets.

Due to an error in communication, during the initial two weeks of HF-based ACF implementation, ACF teams often did not alert cases meeting the suspected case definition that they felt were clearly not EVD (frequently because the case’s symptoms had already resolved by the time of the ACF visit, though the teams may also have considered other criteria such as severity of symptoms or lack of an epidemiological link). A briefing held near the end of the second week emphasized the importance of alerting all such consultations and classifying them as “invalidated” rather than “not alerted”. The proportion of consultations meeting the definition of a suspected case that were alerted increased accordingly: 5% in the first two weeks (Period 1, 23 June-6 July), compared to 96% in the latter 2.5 weeks (Period 2, 7–24 July). More cases were recorded as already alerted during Period 1 (42) than Period 2 (5). This trend was similar but less extreme if counting based on cases recorded in the alerts database rather than ACF investigators’ reporting (41 in Period 1 vs 27 in Period 2).

### Description of alerted cases

Table 2 describes 354 of the 358 total alert cases that had detailed data available in the alert database. The alert cases were young (median age = 12.5) and 42.3% were male. Based on the information recorded in the registers and format of the database, it was not possible to know which symptoms were not present as opposed to not recorded.

**Table 2:** Description of cases alerted by ACF

Variable	Number (%) or Median [IQR] *N* = 354
Demographics	
Age	12.5 years [6–25]
Sex: Male	146 (42.3%)^g^
Symptoms	
Any Bleeding^a^	12 (3.4%)
Spontaneous Abortion	3 (0.8%)
Fever	336 (94.9%)
Headache	194 (54.8%)
Vomiting	179 (50.6%)
Loss of Appetite	191 (54.0%)
Diarrhea	58 (16.4%)
Intense Fatigue	151 (42.7%)
Abdominal Pain	189 (53.4%)
Muscle or Joint Pain	98 (27.7%)
Difficulty Swallowing	4 (1.1%)
Difficulty Breathing	9 (2.5%)
Hiccups	0
Rash	3 (0.8%)
Cough	60 (16.9%)
Weakness	44 (12.4%)
Nausea	62 (17.5%)
≥3 Defining Symptoms from Suspected EVD Case Definition^b^	255 (72.0%)
Documented Symptoms Meet Suspected EVD Case Definition^c^	255 (72.0%)
Diagnoses Recorded	
Malaria	319 (90.1%)
Gastro-intestinal infection^d^	125 (35.3%)
Respiratory infection^e^	23 (6.5%)
Gynecological problem or infection^f^	9 (2.5%)
Unspecified	9 (2.5%)

^a^Any bleeding included: Blood in stool (5 individuals); abnormal vaginal bleeding (4); vomiting blood (1); nosebleed + coughing up blood (1); nosebleed + vomiting blood (1)

^b^Includes all symptoms from “headache” to “rash”, i.e.: headache, vomiting, lack of appetite, diarrhea, intense fatigue, abdominal pain, muscle or joint pain, difficulty swallowing, difficulty breathing, hiccups, and rash

^c^Includes any bleeding, any spontaneous abortion, or fever + ≥3 defining symptoms

^d^Includes those described as gastroenteritis, intestinal parasite infection, typhoid, dysentery, amebiasis, or salmonellosis

^e^Includes those described as respiratory infections, pneumonia, bronchitis, tuberculosis, or influenza-like illness

^f^Includes gynecological infection, threatened abortion, spontaneous abortion, and malaria infection during pregnancy

^g^Nine (2.5%) individuals missing data on sex were removed from the denominator

All alert cases should have met the definition for a suspected EVD case. Using the symptoms available in the database, we were able to reconstruct this for 72.0% of alerted cases. It was not clear whether the remaining cases did not in fact meet the definition or did not have all symptoms recorded. Twelve (3.4%) alerted cases had any bleeding, most frequently abnormal vaginal bleeding (*n* = 4) or blood in the stool (*n* = 5), and 3 (0.8%) had spontaneous abortion. Fever was the most frequently reported symptom (94.9%). The other most common symptoms, in descending order, were: headache (54.8%), loss of appetite (54.0%), abdominal pain (53.4%), and vomiting (50.6%). The two individuals from the HF-based ACF system who were validated as suspected cases until tested negative were both adult males with multiple abnormal bleeding symptoms, and one was also recognized as a previous contact of an EVD case past the period of observation. Abnormal bleeding was also recognized in the two validated suspect cases identified by alerts outside of the HF ACF system during the same time period.

The alerted cases were attributed to malaria in 90.1% of cases (Table 2). Malaria diagnoses were made clinically, as use of malaria rapid diagnostic tests was discouraged given the risk of contamination and potential for malaria-EVD co-infection [22]. The second most common diagnosis was gastrointestinal infection (often in addition to malaria) at 35.3%. Respiratory infections, gynecological problems, and unspecified illnesses accounted for < 10% each. No unexplained deaths were uncovered through the HF-based ACF system.

### Description of visited HFs

The compiled master list of HFs included 140 HFs, of which 104 were initially listed and prioritized, and 36 were added following an ACF visit. Twenty-seven of these 140 HFs were never visited, 11 because they were only accessible by the river, while others for unknown reasons. The greatest number of visits per HF was 14, to a medium-priority HF.

Table 3 summarizes the characteristics of all the HFs from this master list. High-priority HFs were visited most often, followed by medium, and finally low, but some individual lower-priority HFs were visited more often than some individual higher-priority HFs. There were fewer public HFs than private HFs, but these were more frequently high-priority (22/25 high-priority HFs were public, compared with 33/140 HFs overall), and were visited more often by ACF teams than private HFs.

**Table 3:** Description of HFs targeted for ACF in Mbandaka

Variable		Number of HFs	Median Number of Visits Per HF [IQR]
All HFs	N/A	140	2 [1–5]
Type of HF	Private	107	2 [0.5–3]
	Public	33	6 [5–7]
HF Priority	Low	73	1 [0–3]
	Medium	42	3 [2–5]
	High	25	6 [5–7]
Health Zone within city of Mbandaka	Wangata	72	3 [1–4]
	Mbandaka	45	2 [1–5]
	Bolenge	23	3 [0–5]
River Accessibility Only	No	129	3 [1–5]
	Yes	11	0 for all

The targets for number of visits per HF (8 for high-priority, 4 for medium, and 2 for low) were met by 54 (39%) HFs: 6/25 (24%) high-priority HFs, 18/42 (43%) medium-priority HFs, and 30/73 (41%) low-priority HFs.

### Impact on HF EVD awareness

In assessing the impact of HF-based ACF visits on HF EVD awareness, we excluded the first two days of HF-based ACF (23–24 June), as 18/23 ACF visits during this period were missing data on EVD awareness. However, we continued to include these days when counting the number of previous ACF visits to a given HF. The remaining 30 days included 384 HF visits, of which 292 (76%) demonstrated good EVD awareness, 87 (23%) did not demonstrate good EVD awareness, and 5 (1%) values were missing. Good awareness was defined as positive responses to all four EVD awareness indicators (see Methods).

Table 4 shows the results of univariate and multivariate logistic regression with an outcome of good EVD awareness and a random effect for HF. All variables in the table were included in the multivariate model. In univariate and multivariate analysis, awareness of EVD and the alerts system was positively associated with an increasing number of previous ACF visits to the same HF. EVD awareness was also positively associated with the visit occurring later during the HF-based ACF period (in weeks). The association with number of previous visits remained consistent when considering different versions of the variable for week of visit, including dichotomizing to two weeks of HF-based ACF and putting each week separately in the model.

**Table 4:** Univariate and multivariate associations with “good awareness of EVD”

Variable		HF Visits	Visits with Good EVD Awareness	Univariate Odds Ratio	Univariate *P*-Value	Multivariate Odds Ratio	Multivariate *P*-Value
Previous ACF Visits to HF	No, First Visit	90	33	Ref	Ref	Ref	Ref
	Yes, 2nd or 3rd Visit	152	121	11.2 (5.1, 29.3)	< 0.0001	4.4 (2.0, 10.8)	0.0005
	Yes, 4th + Visit	142	138	141.5 (36.4, 830.9)	< 0.0001	15.0 (3.5, 84.3)	0.0007
Week	Continuous	384	292	4.4 (3.0, 7.2)	< 0.0001	2.6 (1.8, 4.2)	< 0.0001
Type of HF	Private	196	130	Ref	Ref	Ref	Ref
	Public	188	162	3.6 (2.0, 7.1)	< 0.0001	2.4 (0.7, 9.5)	0.19
HF Priority	Low	112	74	Ref	Ref	Ref	Ref
	Medium	135	104	1.8 (0.9, 3.4)	0.07	1.0 (0.4, 2.6)	0.92
	High	137	114	3.0 (1.5, 6.7)	0.002	0.6 (0.1, 2.5)	0.46
Health Zone	Wangata	210	146	Ref	Ref	Ref	Ref
	Mbandaka	119	101	2.5 (1.4, 4.7)	0.002	4.1 (1.7, 10.9)	0.002
	Bolenge	55	45	2.4 (1.1, 5.7)	0.03	3.1 (0.9, 11.2)	0.06

Awareness of EVD was greater for HFs in Mbandaka and non-significantly greater for HFs in Bolenge Health Zone, compared to Wangata. Public HFs and high-priority HFs were associated with greater awareness in univariate but not multivariate analyses.

## Discussion

In this paper we summarize the HF-based ACF system set up in Mbandaka, DRC, in the final stages of the Équateur EVD epidemic. The results illustrate both the strengths and challenges of deploying such a system for EVD surveillance and control.

Over the course of 32 days, ACF teams reviewed over 37,000 consultations that took place beginning one week prior to implementation of this system. In total, ACF teams identified 690 consultations meeting the definition of a suspected EVD case, the vast majority of which were not already alerted by the HF. Although previous EVD epidemics have resulted in decreases in healthcare-seeking behavior [23], the low number of confirmed EVD cases in the city of Mbandaka made this less likely. Furthermore, an MoH policy that made healthcare free for the duration of the epidemic likely encouraged people with symptoms to seek care, making this system an effective means of finding individuals meeting the suspected case definition.

A strength of this system was its positive impact on HF awareness of EVD, including knowing the definition of a suspected EVD case and the phone number of the EVD alerts system. HF awareness of EVD improved significantly according to the number of previous ACF visits, an effect that could not be fully explained by general time trends. ACF teams also identified HFs not on the official master list, thus allowing more HFs to benefit from this intervention. On the other hand, the frequency of ACF visits to each HF was highly variable and frequently did not meet pre-specified targets. Initiating analyses of ACF visits early into its implementation, rather than after its completion, could help refine such targets to ensure they are reasonable and on track to being achieved.

One clear challenge of HF-based ACF for EVD shown by these results is the non-specificity of the EVD suspected case definition in this setting. Mbandaka is a large city with a high burden of infectious diseases, and the HF-based ACF system was implemented at a late stage of the epidemic. As seen by the low number of validated alerts, ACF investigators used not only the clinical case definition, but also epidemiological factors, clinical evolution, and general clinical judgement to decide whether an alert should be validated. The inclusion of these other factors in the assessments was necessary given the low specificity of the EVD suspected case definition, the heavy procedures involved in testing each validated suspected case (transfer to ETU, etc), and the low prior probability of EVD given the status of the epidemic. However, this was largely done on an ad-hoc basis, and the subjectivity of these decisions could have increased the risk of an ACF investigator mistakenly invalidating a true EVD case. Similar ACF systems would be strengthened by clear guidelines on how to assess such patients, including specific accounting for clinical improvement, and/or development of safe and effective rapid EVD diagnostics that could be used directly by ACF teams [24, 25]. We also recommend that future ACF systems collect data on the specific reason each alert was validated or invalidated to allow real-time assessments of these decisions.

Another concerning finding was that the vast majority of consultations that met the definition of a suspected EVD case were not alerted by the HFs. In fact, this proportion appeared to decrease over time despite improvements in EVD knowledge. Perhaps this finding could be explained by the number of non-EVD cases who met the suspected EVD case definition. HFs may have been unwilling to spend time and effort to alert cases meeting this definition when they believed their symptoms could be explained by other causes. A lack of trust in the case definition could thus have weakened the alert system and increased the need for HF-based ACF. It is also possible that the HF-based ACF system itself directly decreased the willingness of HFs to spontaneously alert suspected EVD cases, either because the HF staff felt less responsibility for alerting cases or because they realized that the majority of alert cases uncovered by the ACF teams were not validated as suspected cases and did not, in fact, have EVD.

Ideally, future HF-based ACF systems would focus on both improving HF EVD knowledge and encouraging HFs to alert cases on their own. Earlier implementation of HF-based ACF during future EVD epidemics could leverage the positive impacts of ACF on HF awareness to train HCWs regarding proper utilization of the alerts system. As the alert system becomes more effective, the HF-based ACF system would then contribute less to the discovery of suspected cases, though it could still be used periodically to monitor the performance of the alert system and reinforce HCW awareness. However, earlier implementation would also create greater resource requirements. In this outbreak, HF-based ACF was implemented after the end of contact tracing by the same teams of people who had previously been investigating contacts. Given the low positive predictive value of HF-based ACF seen here and the high effectiveness of contact tracing in stopping EVD epidemics, HF-based ACF should only be considered earlier in the outbreak if sufficient resources exist for both interventions; it is important that resources for contact tracing not be diverted towards HF-based ACF. Future surveillance systems could also consider integrating additional assessments into ACF visits to increase their utility, such as of supplies of personal protective equipment and knowledge of infection prevention and control [26], and connecting HFs with the appropriate resources as necessary.

Approximately 90% of all alert cases found through HF-based ACF were clinically attributed to malaria. Previous studies have shown high prevalence of malaria in Bolenge Health Zone [27], and the young ages of alerted cases (median 12.5 years) is also consistent with a high burden of malaria. During the West African EVD epidemic, mass drug administration for malaria was implemented in some areas of Sierra Leone to reduce the difficulty in identifying EVD cases and led to significant decreases in the number of alerts [28]. This approach was not considered necessary during the Équateur outbreak, but could be considered in future EVD epidemics in hyperendemic malaria areas. Indeed it has been implemented in Beni during the most recent DRC epidemic [29]. The large burden of illness attributable to malaria and other diseases also demonstrates the need for public health investment and research outside of emergency situations in poorly resourced settings such as Mbandaka.

The limitations of this paper reflect the challenges of EVD surveillance in Mbandaka. The data were collected for public health purposes and, given competing resource priorities, not collected with the same degree of quality assurance as for a research study. However, we revisited the completed paper forms and compared the available databases to check for and correct discrepancies before the analyses presented here. The diagnoses recorded for alert cases were made clinically and typically without confirmatory testing. Given the high burden of malaria in this setting, it is likely that malaria tests would have been positive even for illnesses with other causes. Finally, some individuals meeting the suspected case definition may have been missed due to incomplete reporting in HF registries.

## Conclusion

HF-based ACF may improve HF knowledge of EVD symptoms and the procedure for reporting suspected cases. It can also help detect individuals seeking care for symptoms consistent with the EVD suspected case definition, particularly when the alert system is underperforming. Both of these finding support the roll-out of similar ACF systems during future EVD epidemics, particularly towards the beginning of these epidemics. However, the low positive predictive value of the definition of suspected EVD cases in areas with high burdens of other infectious diseases and low circulation of EVD implies in a heavy reliance on factors outside the clinical case definition to determine which alerts should be validated and transferred to the ETU for EVD testing. Future ACF systems should define clear guidelines about which cases should be validated and tested, based on criteria that may be context-specific, and focus on ensuring all cases are alerted starting from the beginning of the outbreak.

## Supplementary information

**Additional file 1.** Model Details. This model provides the R syntax for the models used to assess EVD awareness (DOCX 21 kb)

## Data Availability

The data sets used and/or analyzed during the current study are available from the corresponding author on reasonable request.

## Abbreviations

ACF: Active Case Finding
ETU: Ebola Treatment Unit
EVD: Ebola Virus Disease
HCW: Health Care Worker
HF: Health Facility
MoH: Ministry of Health
WHO: World Health Organization
