# Supplementary material for: Assessment of a health facility based active case finding system for Ebola virus disease in Mbandaka, Democratic Republic of the Congo, June–July 2018
Source: BMC Infect Dis. 2019 Nov 21;19:981. doi: 10.1186/s12879-019-4600-4 (PMC6873572; doi:10.1186/s12879-019-4600-4)
Supplement: Supplementary file 1 — Additional file 1. Model Details. This model provides the R syntax for the models used to assess EVD awareness (DOCX 21 kb) [file 12879_2019_4600_MOESM1_ESM.docx]

Additional File 1: Model Details

Assessment of a health facility based active case finding system for Ebola Virus Disease in Mbandaka, Democratic Republic of the Congo, June-July 2018

Amber Kunkel^*^, Mory Keita^*^, Boubacar Diallo, Olivier le Polain de Waroux, Lorenzo Subissi, Bocar Wague, Roger Molala, Pierre Lonfandjo, Sébastien Bokoo Bokete, William Perea, and Mamoudou Harouna Djingarey

*^*^These authors contributed equally*

**Description of Data and Analyses**

Logistic regression models with a random intercept term for health facility (HF) added to account for repeated visits were run to evaluate the effect of various HF and visit characteristics on EVD (Ebola Virus Disease) awareness. This was done using R package lme4 [1].

The dataset dat_visits_2 has a row for each HF-based active case finding (ACF) visit to a health facility.

The variables in the dataset used below are as follows:

- Priorite: HF priority. 1: low priority, 2: medium, 3: high
- week: Week of the visit to the HF, ranging from 1 (first week of HF-based ACF) to 5
- first_two_wk: 1 if the HF visit occurred in the first two weeks of HF-based ACF, 0 otherwise
- Public: 1 for public HFs, 0 for private HFs
- ZS.x: Health zone. Factor variable with levels “BOLENGE”, “MBANDAKA”, and “WANGATA”. “WANGATA” was set to be the reference value.
- HF_ID: ID variable for health facility
- which_visit_cat: How many previous visits to the same health facility? 1: first visit; 2: 2^nd^-3^rd^ visit; 3: 4^th^ or greater visit
- all_yes: 1 if the answer to all four knowledge indicators was “yes” for a given HF-based ACF visit; 0 otherwise

**Models**

*Univariate Model: Previous visits to HF*

glmer(all_yes ~ as.factor(which_visit_cat) + (1| HF_ID),

data= dat_visits_2, family=binomial(link="logit"))

*Univariate Models: Week of Visit*

glmer(all_yes ~ week + (1| HF_ID),

data= dat_visits_2, family=binomial(link="logit"))

*Note: this first model was the primary model reported; the rest were tested as sensitivity analyses and showed consistent results

glmer(all_yes ~ first_two_wk + (1| HF_ID),

data= dat_visits_2, family=binomial(link="logit"))

glmer(all_yes ~ as.factor(week) + (1|HF_ID),

data= dat_visits_2, family=binomial(link="logit"))

*Univariate Model: Public vs Private HF*

glmer(all_yes ~ as.factor(Public) + (1|HF_ID),

data= dat_visits_2, family=binomial(link="logit"))

*Univariate Model: HF Priority*

glmer(all_yes ~ as.factor(Priorite) + (1|HF_ID),

data= dat_visits_2, family=binomial(link="logit"))

*Univariate Model: Health Zone*

glmer(all_yes ~ ZS.x + (1|HF_ID),

data= dat_visits_2, family=binomial(link="logit"))

*Primary Multivariate Model*

glmer(all_yes ~ week + as.factor(Priorite) + Public + ZS.x +

as.factor(which_visit_cat) + (1|HF_ID),

data= dat_visits_2, family=binomial(link="logit"),

control=glmerControl(optimizer="bobyqa"))

*Multivariate Models Tested as Sensitivity Analyses*

glmer(all_yes ~ first_two_wk + as.factor(Priorite) + Public + ZS.x +

as.factor(which_visit_cat) + (1|HF_ID),

data= dat_visits_2, family=binomial(link="logit"),

control=glmerControl(optimizer="bobyqa"))

glmer(all_yes ~ as.factor(week) + as.factor(Priorite) + Public + ZS.x +

as.factor(which_visit_cat) + (1|HF_ID),

data= dat_visits_2, family=binomial(link="logit"),

control=glmerControl(optimizer="bobyqa"))

**Reference**

[1] Bates D, Maechler M, Bolker B, Walker S. Fitting Linear Mixed-Effects Models Using lme4. J Stat Softw 2015;67:1–48. doi:10.18637/jss.v067.i01.
